# Complement pathway changes at age 12 are associated with psychotic experiences at age 18 in a longitudinal population-based study: evidence for a role of stress

**DOI:** 10.1038/s41380-018-0306-z

**Published:** 2019-01-11

**Authors:** Melanie Föcking, Sophie Sabherwal, Hannah M. Cates, Caitriona Scaife, Patrick Dicker, Magdalena Hryniewiecka, Kieran Wynne, Bart P. F. Rutten, Glyn Lewis, Mary Cannon, Eric J. Nestler, Meike Heurich, Gerard Cagney, Stanley Zammit, David R. Cotter

**Affiliations:** 1 Department of Psychiatry, Royal College of Surgeons in Ireland, Dublin, Ireland; 2 Friedman Brain Institute and Icahn School of Medicine at Mount Sinai, NY New York, USA; 3 School of Biomolecular and Biomedical Science, Conway Institute, University College Dublin (UCD), Belfield Dublin 4, Ireland; 4 Department of Epidemiology and Public Health, Royal College of Surgeons in Ireland, Dublin, Ireland; 5 Department of Psychiatry and Neuropsychology, School for Mental Health and Neuroscience, Maastricht University Medical Centre+, Maastricht, The Netherlands; 6 Division of Psychiatry, UCL, London, UK; 7 School of Pharmacy and Pharmaceutical Sciences, Cardiff University, Cardiff, UK; 8 MRC Centre for Neuropsychiatric Genetics and Genomics, Cardiff University, Cardiff, UK; 9 Centre for Academic Mental Health, Bristol Medical School, University of Bristol, Bristol, UK

**Keywords:** Biological techniques, Neuroscience

## Abstract

The complement cascade is a major component of the immune defence against infection, and there is increasing evidence for a role of dysregulated complement in major psychiatric disorders. We undertook a directed proteomic analysis of the complement signalling pathway (*n* = 29 proteins) using data-independent acquisition. Participants were recruited from the UK avon longitudinal study of parents and children (ALSPAC) cohort who participated in psychiatric assessment interviews at ages 12 and 18. Protein expression levels at age 12 among individuals who reported psychotic experiences (PEs) at age 18 (*n* = 64) were compared with age-matched controls (*n* = 67). Six out of the 29 targeted complement proteins or protein subcomponents were significantly upregulated following correction for multiple comparisons (VTN↑, C1RL↑, C8B↑, C8A↑, CFH↑, and C5↑). We then undertook an unbiased plasma proteomic analysis of mice exposed to chronic social stress and observed dysregulation of 11 complement proteins, including three that were altered in the same direction in individuals with PE (C1R↑, CFH↑, and C5↑). Our findings indicate that dysregulation of the complement protein pathway in blood is associated with incidence of psychotic experiences and that these changes may reflect exposure to stress.

## Introduction

The early identification and treatment of subjects with psychiatric disorders, both psychotic and affective, significantly improves their clinical outcome [1]. Thus, over the last decade, there has been a shift in research to focus on the so-called ‘at risk mental state’ (ARMS) or ultra-high risk (UHR) for psychosis [2] with the aim of identifying vulnerable subjects and offering early treatment to prevent psychosis [3, 4]. Even so, only 16–35% UHR subjects go on to convert to psychosis [5, 6], with 50–65% of these subsequently experiencing non-psychotic mental disorders, such as depression and anxiety [2, 7]. Consequently, there is now an increasing focus not just on the vulnerability to psychotic disorder represented by psychotic experiences but on vulnerability to major psychiatric disorders generally.

Blood-based studies of the ARMS and UHR, focusing on inflammation markers, have been undertaken and have shown largely consistent changes implicating a pro-inflammatory process in both psychosis and affective disorder [8, 9]. These findings are supported and extended by discovery proteomic studies of first episode psychosis and schizophrenia implicating the acute-phase response, glucocorticoid receptor signalling, coagulation, and lipid and glucose metabolism [10, 11]. Furthermore, inflammatory cytokines, chemokines, and growth factors have been assessed in the blood during the perinatal periods and during childhood in subjects who subsequently developed schizophrenia, and in those with a first episode psychosis [12–16]. Together these studies demonstrated a picture of enhanced inflammatory tone during and preceding psychosis, and indeed other major mental illnesses [17]. Whereas the basis of these changes is not clear, numerous risk factors for schizophrenia, such as genetic background, but also exposure to abuse, maternal stress during pregnancy, prenatal famine, obstetric complications, exposure to infectious agents, or alterations in the microbiome and adolescent cannabis use have all been described and hypothesised to lead to raised inflammatory tone [18–20]. Post-mortem brain studies support the evidence for a role of inflammation, suggesting that this process is involved during early and later stages of the disorder [21–23].

Previous studies based on  the ALSPAC cohort, a prospective general population cohort based in the Bristol area in South West England, have shown subgroups of subjects who developed psychotic disorder (PD) and psychotic experiences (PEs) [24] at age 18. These groups showed alterations in cortical white matter microstructure [25], working memory [26], and raised inflammatory markers in childhood [12] in subjects  with PE at age 18. We  recently used discovery methods to compare the plasma proteome of age 12 subjects who developed psychotic disorder at age 18 and we found evidence implicating some protein members of the complement pathway at age 12 in subjects with PD at age 18 [27]. The complement system [28–30] has been implicated previously in schizophrenia and other major psychiatric disorders [31, 32]. Complement has very well described roles in inflammation both peripherally and in the brain, roles in plasticity, neuronal growth, and neuroprotection are increasingly appreciated (for review see [33–35]). The genetic contribution of complement component 4 (C4) to schizophrenia has been reported and a contribution to schizophrenia risk through the regulation of synaptic plasticity [23] and cortical thinning is proposed [36, 37].

The current study had two aims, first, we used targeted proteomic methods to carry out a comprehensive analysis of the complement pathway within the plasma of  age 12 subjects who reported psychotic experiences at age 18. Second, due to the known relationship between exposure to stress and later psychosis [38], we also examined the plasma proteome of mice exposed to chronic social stress. The findings of this study are relevant to our understanding of the role of the complement system in vulnerability to major adult psychiatric disorder outcomes.

## Methods

For extended materials and methods, please refer to Supplementary Methods.

### Participants

The ALSPAC cohort is a prospective population-based cohort, and a rich resource of demographic, environmental, and clinical data on the individuals involved [39, 40]. Written informed consent was obtained prior to taking the plasma samples. The case and control samples were retrieved from the ALSPAC archive at the same time, stored under the same conditions, and tested in a “blinded” fashion where samples from the test groups were admixed. The asymptomatic controls were derived from a random selection of all the participants, who provided plasma samples, and who did not have PEs at either age, 12 or 18. Ethical approval for the study was obtained from the ALSPAC Ethics and Law Committee and the Local Research Ethics Committee (REC1240). Please note that the study website contains details of all the data that are available through a fully searchable data dictionary (http://www.bristol.ac.uk/alspac/researchers/access).

### Measures of psychotic experiences

Psychotic experiences (PEs) were identified at 12 and 18 years through face-to-face, semi-structured Psychosis-Like symptom (PLIKS) interviews [24], conducted by trained psychology graduates in assessment clinics, and were coded according to the definitions and rating rules for the Schedules for Clinical Assessment in Neuropsychiatry, Version 2.0 (Organisation 1994 Interviewers rated PEs as not present, suspected or definite). The psychotic experiences (PE) group comprised subjects who fulfilled criteria for definite  PEs [24] at age 18, but not age 12.

### Study design

We undertook a nested case-control study from individuals with plasma samples available at age 12 we selected all subjects who had definite psychotic experiences at age 18 but not at age 12 (*n* = 64). Age-matched controls were randomly selected from individuals with available plasma samples at age 12 who did not have either suspected or definite PEs at ages 12 or 18 (*n* = 67).

See Table 1. With regard to psychotropic drug use, 5 of the 64 subjects with PEs at age 18 were recorded as taking psychotropic medication at age 18. No subjects reported psychotropic drug use at age 12.

**Table 1:** Descriptive information for ALSPAC subjects

	Psychotic experiences (PE) study	
	Cases (PE12 = 0, PE18 = Def)	Controls (PE12 = 0, PE18 = 0)
Proteomics study	64	67
Gender	36 F, 28 M	28 F, 39 M
BMI at age 12 Mean (Std Dev)	18.96 (2.88)	17.72 (2.52)
Ethnicity	57 W, 3 NW, 4 NA	64 W, 3 NA
Pliks at age 18	64 definite	None
Social economic status	28 NM, 30 M, 6 NA	45 NM, 17 M
Depression at age 18	20 ND, 39 D, 5 NA	58 ND, 9 D
Received medication for hallucinations/delusions at age 18	5 yes	NA

For gender *F* Female, *M* Male. Body mass index (BMI) at age 12 is reported, where missing BMI variables were replaced with the mean according to gender. For ethnicity— white, *NW* non-white, *NA* missing. PLIKS at age 12 and age 18 are reported, however in this analysis we used PLIKS at age 18 as the main outcome measure for our proteomic analysis. For Depression created a binary outcome: individuals with CIS-R scores >7 as depression (D) and <7 as no depression (ND)

### Blood collection

For all ALSPAC participants, blood samples from non-fasting individuals were collected at ~12 years of age. Blood was collected in 7.5 ml Plasma Lithium-Heparin S-Monovette tubes (Sarstedt). Once collected, samples were stored on ice for a maximum of 90 min until processed. After centrifugation, the plasma was stored in aliquots at −80 °C. All samples underwent a single freeze thaw cycle to allow aliquotting prior to the study. The standard quality of the plasma samples was ensured by assessing the overall MS protein profile to facilitate the identification of outlier protein expression profiles (see Supplementary Figure 1a and b for the PE and the PPE, respectively).

### High-abundance protein depletion of plasma samples

To improve the dynamic range for proteomic analysis, 40 µl of plasma from each case in all samples was immunodepleted of the 14 most abundant proteins (Alpha-1-antitrypsin, A1-acid glycoprotein, Serum Albumin, Alpha2-macroglobulin, Apolipoprotein A-I, Apolipoptrotein A-II, Complement C3, Fibrinogen alpha/beta/gamma, Haptoglobin, IgG A, IgG G, IgG M, Transthyretin, and Serotransferrin), using the Agilent Hu14 affinity removal system (MARS) coupled to a high-performance liquid chromatography (HPLC) system [41] (see Supplementary Methods).

### Sample preparation for mass spectrometry

Protein digestion and peptide purification were performed as previously described [42], and is further detailed in Supplementary Methods.

### Proteomic analysis of PE focusing on complement pathway

We used the semi-targeted approach of data-independent acquisition (DIA) to target 29 members of the complement pathway as defined by KEGG pathway analysis (http://www.genome.jp/kegg/pathway.html) and see Supplementary Table 1. DIA overcomes many of the limitations of untargeted proteomics, for example missing values [43–46]. For DIA in the PE and the PPE studies, 5 μl of each sample was injected into the Thermo Scientific Q-Exactive, connected to a Dionex Ultimate 3000 (RSLCnano) chromatography system, and data were acquired in DIA mode.

The DIA isolation scheme and multiplexing strategy was based on that from Egertson et al., in which five separate 4-*m*/*z* isolation windows are analysed per spectrum [47, 48]. In order to create a spectral library for targeted chromatogram extraction, we used an internal standard for quality control (QC), where an equal aliquot from each protein digest in the experiment was pooled into one sample for use as an internal QC. QC samples were injected in data-dependent acquisition (DDA) mode and was injected three times at the beginning of the MS study to condition the column, and subsequently after every 10 injections throughout the experiment to monitor the MS performance. To facilitate accurate prediction of peptide retention calculation in Skyline^TM^ for DIA data, protein digests were spiked with the Pierce^TM^ Peptide Retention Time Calibration Mixture (4 fmol/μl), according to the manufacturers’ instructions (see Supplementary Figure 2A and B for extensive quality control). Data used for this submission will be made available on request to the ALSPAC  Executive Committee (alspac-exec@bristol.ac.uk).

### Social Defeat Stress Mouse Model

We used a well-established animal model of chronic social defeat stress [49, 50]. Male, 8-week-old C57BL/6J mice were exposed to 10 consecutive days of 5-min defeats by a novel CD1 aggressor mouse and were then housed across a Plexiglas divider to allow for sensory contact for the remainder of the day. Mice susceptible to this repeated stress were identified by their avoidance of interaction with a novel mouse 24 h after day 10 of defeat in a social interaction test. Animals (*n* = 5 stressed and *n* = 5 control mice) were killed on day 30 and trunk blood was obtained for analysis [51].

#### Protein depletion of mouse plasma samples

To improve the dynamic range for proteomic analysis, 40 µl of plasma from each animal was immunodepleted of the three most abundant proteins (Albumin, IgG, Transferrin) using the Multi Affinity Removal Column Mouse-3 (Agilent Technologies, UK) coupled to a HPLC system [41]. For more details of the animal model, sample preparation, and mass spectrometry, please see Supplementary Methods.

### Bioinformatics and statistical analysis

#### Semi-targeted analysis of complement pathway proteins in PE

All DIA files from the PE study were  analysed in Skyline (V3.5.0; https://skyline.gs.washington.edu), as detailed by Egertson et al. [47, 48]. We identified and quantified all proteins and their peptides listed as contributing to the complement pathway according to KEGG (http://www.genome.jp/kegg/pathway.html)). For a full list of the fragments targeted and quantified, please refer to Supplementary Tables 2a and 2b. All peptides and associated fragment ions were visually checked in all samples, and peak editing was undertaken where necessary (for details see Supplementary Documents). Pre-processing and statistical analysis of the fragment-level data were undertaken in mapDIA [46].

As there were differences between the PE group cases and controls in terms of BMI and gender we co-varied for these variables in our analyses. There were no significant differences between the groups in other variables as listed in Table 1, and therefore we did not correct for these potential confounders. The demographic and clinical data were tested for differences between case and control group using the Fisher’s Exact test and the two-sample sample *t*-test. Statistical significance was determined at the 5% level of significance. Comparison of complement pathway proteins between groups was performed using a false discovery rate (FDR) of 5%, as described by Benjamini-Hochberg [52].

### Social Defeat Stress Mouse Model

The bioinformatics and statistical analysis of the animal model of stress was undertaken using the MaxQuant programme specifically for label-free experiments using high resolution instruments supported by Andromeda as a database search engine for peptide identification [53]. Raw LFQ intensities were extracted from the MaxQuant software and log base 2 transformed prior to analysis to eliminate distributional skew and to give approximate normality. To avoid bias associated with protein under-representation between groups, proteins were excluded in cases where there was less than 80% availability of the LFQ intensities in each biological group. After data filtering, 704 LFQ values remained.

The significance level was calculated following correction according for FDR [52] based on the whole-discovery proteome (*n* = 262), but, because, the focus of the study is on the complement pathway proteins we only report on these latter proteins.

## Results

### PE study

Two PE cases and no controls were excluded from the bioinformatics analysis due to poor chromatographic profiles.  The final analyses compared the ALSPAC subgroup of participants with PE (*n* = 64), at age 18 to controls (*n* = 67; Table 1).

**Fig. 1:**
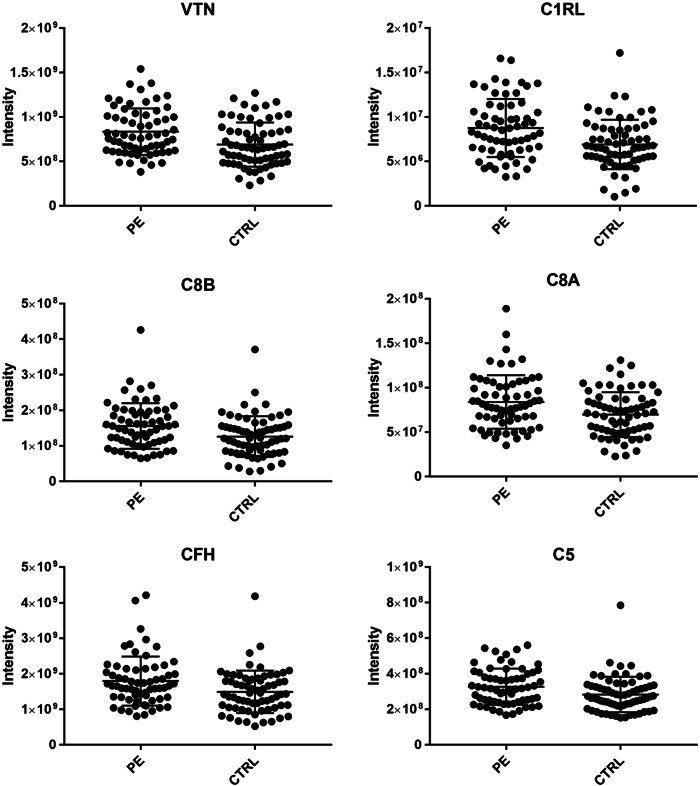
Plots the protein abundances derived from the mass spectrometry data for the significantly regulated proteins, VTN, C1RL, C8B, C8A, CFH, and C5

The semi-targeted DIA approach (see Supplementary Material) was used to quantify the levels of 29 complement pathway proteins in the PE group. Each of the 29 complement pathway proteins had peptides suitable for this analysis (Supplementary Tables 2a and 2b) and following adjustment for gender and BMI (see Table 2). Among these, we observed differential expression in eight proteins (VTN, C1RL, C8B, C8A, CFH, C5, C4BPA, and C2) with six proteins remaining significant following correction for multiple comparisons: VTN (*p* < 0.0005)↑,C1RL (*p* < 0.0005)↑, C8B (*p* < 0.005)↑, C8A (*p* < 0.01)↑, CFH (*p* < 0.01)↑, and C5 (*p* < 0.01)↑. (See Table 2 for all protein level results and Figure 1 for the protein abundance from the Mass spectrometry data for the significantly regulated proteins.)

**Table 2:** Differential protein expression in PE

		PE study findings		
Protein names	Gene names	Fold change	*p*-value	FDR
Vitronectin	*VTN*	**1.219**	**0.00150**	**0.02226**
Complement C1r subcomponent-like protein	*C1RL*	**1.291**	**0.00154**	**0.02226**
Complement component C8 beta chain	*C8B*	**1.270**	**0.00352**	**0.03399**
Complement component C8 alpha chain	*C8A*	**1.207**	**0.00535**	**0.03537**
Complement factor H	*CFH*	**1.207**	**0.00610**	**0.03537**
Complement C5	*C5*	**1.161**	**0.00847**	**0.04096**
C4b-binding protein alpha chain	*C4BPA*	−1.155	0.02734	0.11326
Complement C2	*C2*	1.124	0.03709	0.13446
Mannan-binding lectin serine protease 1	*MASP1*	1.193	0.05725	0.18448
Complement C1s subcomponent	*C1S*	1.118	0.07756	0.22036
Complement factor B	*CFB*	1.103	0.08624	0.22036
Complement component C8 gamma chain	*C8G*	1.162	0.09791	0.22036
Complement C1q subcomponent subunit A	*C1QA*	1.139	0.09878	0.22036
Complement C4-A	*C4A*	1.122	0.11874	0.24596
Complement C1q subcomponent subunit B	*C1QB*	1.251	0.15870	0.30683
Complement factor I	*CFI*	1.094	0.20871	0.36678
Clusterin	*CLU*	1.097	0.21501	0.36678
Complement C1r subcomponent	*C1R*	1.095	0.23435	0.37756
Complement component C6	*C6*	1.078	0.28313	0.43214
Complement C1q subcomponent subunit C	*C1QC*	1.068	0.32245	0.46611
C4b-binding protein beta chain	*C4BPB*	−1.087	0.33762	0.46611
Complement factor H-related protein 5	*CFHR5*	−1.189	0.35984	0.46611
Complement component C9	*C9*	1.066	0.36968	0.46611
Complement factor D	*CFD*	1.235	0.38710	0.46774
Plasma protease C1 inhibitor	*SERPING1*	1.106	0.41419	0.48046
Mannose-binding protein C	*MBL2*	1.069	0.61155	0.68211
Complement C4-B	*C4B*	1.077	0.73548	0.78996
Complement component C7	*C7*	1.023	0.78675	0.81485
Complement C3	*C3*	−1.018	0.84266	0.84266

Semi-targeted proteomic analysis of 29 biomarker candidates between cases (*n* = 64) and controls (*n* = 67) in the PE cohort. Protein level data were assessed for significance between the PE cases and healthy controls, following correction for False Discovery as described by Benjamini-Hochberg [52], and following adjustment for BMI and gender, respectively. The protein name, gene name, fold change (FC) in disorder, ANCOVA adjusted *p*-values, and FDR cutoff values are listed for all 29 proteins profiled. Proteins are sorted by *p*-value for the PE study. The FDR positive findings are depicted in bold

### Social Defeat Stress Mouse Model

We found 10 complement proteins differentially expressed following FDR adjustment for the 19 complement proteins quantified; seven complement proteins were upregulated (CFH, CFI, C5,C4BP, C1QB, C3, and C1r) and three were downregulated (C9, C8G, and C4b). See Supplementary Table 3 for detailed results.

## Discussion

Our study provides evidence that altered expression of plasma complement proteins at age 12 is associated with psychotic experiences (PE) at age 18. Because, subjects who report psychotic experiences (PEs) are at increased risk not solely for schizophrenia, but for other major psychiatric disorders, such as depression and anxiety disorders [2, 7], our findings are of broad relevance to adult psychiatric syndromes. The complement pathway has recently been highlighted as important in schizophrenia from genomic, neuroimaging, and biomarker studies, and over the last decade, its importance in inflammatory and degenerative brain disorders has been increasingly appreciated [36]. Thus, our study represents a further step in our understanding of the involvement of the complement pathway in disease and suggests that alterations in this pathway as early as age 12 are associated with psychotic experiences and thus vulnerability to later psychiatric disorders generally. Measures of complement pathway protein expression should be considered for inclusion in future psychosis risk prediction studies, such as those using measures from numerous various diverse domains, such as neuropsychology, neuroimaging, and clinical phenotype [4, 6].

Using a unique prospective cohort, we first investigated blood plasma samples obtained from children at age 12 who reported psychotic experiences (PE) at age 18. We specifically studied 29 members of the complement protein pathway and following correction for multiple comparisons observed six proteins to be upregulated (VTN, C1RL, C8B, C8A, CFH, and C5). These findings from the ALSPAC cohort relating complement changes associated with PE, confirm and extend the findings from our previous smaller discovery proteomic study of age 12 protein biomarkers of psychotic disorders at age 18 [27], in which we also observed significant elevations of CFH and VTN and reductions in C4BPA and C4BPB. In contrast to the previous paper, the current study is larger  (64 cases vs 38) and focused on the entire set of 34 complement proteins (versus 9) of which we successfully targeted 29. We also tested our findings in an animal model of sociala defeat stress to investigate possible etiological mechanisms.

Our findings of altered expression of complement pathway proteins implicates both the classical (C1RL, C2, and C4bp) and terminal (C8, C9) pathway, but also suggest an involvement of the alternative pathway (CFH, CFD). The involvement of the classical pathway in schizophrenia has been previously suggested [30, 54]; and was recently confirmed through the genetic association of classical pathway component C4 [23]. One recent study found decreased TCC complex (sC5b-9) plasma levels, a marker of terminal pathway activation in patients with first episode psychosis [55]. Another study showed increased alternative pathway activity in schizophrenia patients [56]. Our data point to the general enhancement in complement activity as a result of increased complement component levels in plasma.

Overall, the complement and coagulation cascade has previously been identified as the most significant pathway implicated in plasma samples of drug naive schizophrenia patients [10, 28, 41, 55–59]. The cause of these changes are not known, but are in keeping with evidence for raised inflammatory tone preceding psychosis and major psychiatric disorders generally [8, 12–17, 60]. The complement system is tightly functionally interlinked with inflammatory cytokines and chemokines with which it reciprocally interacts [33, 35]. Diverse early and later life stresses are associated with raised inflammatory measures, such as IL6, TNFα, and CRP and thus these factors may have roles in the complement changes that we observe [18]. However, increased complement activity itself may be primarily responsible for the raised inflammatory tone observed in major psychiatric disorders and not merely a reflection of that process. This possibility is supported by the vulnerability associated with genetic variation of complement 4 [23] and will have implications for  potential complement-based therapeutics [61] of at risk children demonstrating elevated complement activity.

In order to investigate the potential role of exposure to psychosocial stress in the observed plasma complement pathway changes, we undertook an unbiased (DDA) proteomic study of the plasma of mice exposed to chronic social defeat stress. Complement protein changes were prominent in the plasma of these mice, showing increases in seven (CFH, CFI, C5, C4bpa, C1qb, C1R, and C1qc) and reductions in four complement proteins (C9, C8g, C4b, and SerpinG). These clearly indicate that dysregulated complement pathway protein expression is associated with exposure of adult mice to psychosocial stress. To our knowledge this is the first such study of complement proteins in  social stress. Previously, a single study of heat stressed cows has observed complement activation [62]. Our psychosocial stress study shows interesting overlaps with the results of the PE study in demonstrating upregulated CFH, CFI (alternative pathway) and C1 complex subcomponent C1R (classical pathway). In contrast, social defeat is associated with reduced C9 and C8g (terminal pathway) and in the PE study these latter proteins were increased. Interestingly, the decrease in terminal complement components C9 and C8 in mice is mirrored in a recent study showing decreased TCC in first episode psychosis (FEP) compared to normal controls [55]. This suggests a distinct mechanism for FEP, with FEP potentially showing a closer association to proximate psychological stress, which likely reflects a more acute inflammatory response, and is somewhat distinct from the lower grade upregulated inflammatory tone we observe to be associated with future psychotic experiences. Future studies are needed to address the distinct complement pathway changes associated with stress at different developmental time points and following different recovery periods.

The complement system within the brain has important functions in the regulation of synaptic plasticity [23, 34, 35, 63] and cognitive function [64] and is associated with brain disorders [61, 65–67]. Our findings are thus in keeping both with the literature implicating inflammation in major psychiatric disorders [18, 68] and with potential mechanisms involved in complement and altered synaptic plasticity [20, 23, 36, 69–71]. It is not yet clear, however, to what extent peripheral complement pathway changes such as we have observed are reflected within the brain. Under normal physiological conditions the majority of plasma complement proteins are believed to be produced within the liver [72], and C1q in the brain is produced specially in microglia [73]. However, it is not known if this is altered under stress or situations where the blood brain barrier may be compromised. Of interest, stress is associated with increased microglial activity in the brain [74] and increased microglial activity and numbers are reported in schizophrenia [22, 75–78]. This raises the intriguing possibility that stress-induced complement activation may mediate increased microglial activity and synaptic plasticity in schizophrenia and other major psychiatric disorders. Future studies will need to investigate the relationship between peripheral and central complement pathway activation, microglial function, and synaptic plasticity. Furthermore, considering the longitudinal relationship that we show between complement pathway proteins and psychosis the long-term consequences of altered peripheral complement pathway, and its manipulation, will need to be assessed in the brains of animal models of psychiatric disorders. A testable hypothesis with the potential for clinical translation, is that complement 1 inhibition may protect from stress-induced contribution to psychotic experiences and psychotic disorder.

Our study is not without its limitations. First, our study utilised the uniquely characterised ALSPAC cohort and we could not access a similar age-matched sample in which we could perform a direct replication of the PE study. However, the findings of the PE study overlap with the findings in PD [27] that we observed previously in terms of C4BPA, CFH, C1R, and VTN. Both  studies show a general upregulation of complement protein expression, and they both overlapped with the animal stress study in showing upregulation of CFH and C1R. A second limitation of our study is that we do not have long-term outcome data and thus the more precise long-term psychiatric outcomes of psychotic experiences at age 18 cannot yet be analysed in the context of complement pathway protein expression. Third, whereas various approaches for bioinformatic analyses of DIA datasets are still under development [46], we used a conservative method for the analysis of DIA data which generated protein level intensities from peptide fragment-level data. Fourth, we are able to report fold changes as low as 1.16 as significant, because our study is relatively well powered. However, these small effect sizes have obvious implications for the practicality of using such markers for screening. Fifth, we controlled for BMI and gender in our analyses due to known effects of these variables on inflammatory marker expression [79, 80]. However, both BMI and gender can themselves impact on mental health and psychopathology [81, 82] and this can be considered in future studies. Finally, the social defeat model, whereas a well-established model of exposure to stress, is not a standard model of psychosis. Future work studying the involvement of complement pathway and indeed the impact of its inhibition in for example a double-hit animal model (e.g., [83]) are planned.

In conclusion, our study is unique in focusing on the entire plasma complement pathway proteins at age 12 associated with PEs at age 18. Our study provides evidence for alterations in the complement pathway among subjects with PEs and following exposure to social stress in mice. Future studies are needed to elaborate further on our understanding of the cause and the consequence of these changes and whether the complement pathway represents a drug-able target for future psychiatric illness among children who present with psychotic experiences.

## Electronic supplementary material

Supplementary Methods

Supplementary Figure 1

Supplementary Figure 2

Supplementary Table 1

Supplementary Table 2

Supplementary Table 3
