# Supplementary material for: Complement pathway changes at age 12 are associated with psychotic experiences at age 18 in a longitudinal population-based study: evidence for a role of stress
Source: Mol Psychiatry. 2019 Jan 11;26(2):524–33. doi: 10.1038/s41380-018-0306-z (PMC6906256; doi:10.1038/s41380-018-0306-z)
Supplement: Supplementary file 1 — Supplementary Methods [file 41380_2018_306_MOESM1_ESM.docx]

**SUPPLEMENTARY METHODS**

**High-Abundance Protein Depletion of Human Plasma Samples**

To improve the dynamic range for proteomic analysis, 40µl of plasma from each case was immunodepleted for removal of the 14 most abundant proteins (Alpha-1-antitrypsin, A1-acid glycoprotein, Serum Albumin, Alpha2-macroglobulin, Apolipoprotein A-I, Apolipoptrotein A-II, Complement C3, Fibrinogen alpha/beta/gamma, Haptoglobin, IgG A, IgG G, IgG M, Transthyretin, and Serotransferrin) using the Agilent Hu14 Affinity Removal System (MARS) coupled to a High Performance Liquid Chromatography (HPLC, Shimadzu LC-10AT) system [[1](#_ENREF_1)]. Protein depletion was undertaken according to the manufacturer’s instructions and buffer exchange was performed with 50mM ammonium bicarbonate using spin columns with a 10kDA-molecular weight cut-off (Merck Millipore). Prior to sample preparation for mass spectrometry, the protein concentration was determined using a Bradford Assay [[2](#_ENREF_2)], according to the manufacturer’s (BioRad) instructions.

**Sample Preparation for Mass Spectrometry**

Protein digestion and peptide purification was performed as previously described [[3](#_ENREF_3)]. For quality control (QC), an equal aliquot from each protein digest in the experiment was pooled into one sample for use as an internal QC. This QC standard was injected at the beginning of the MS study to condition the column, and after every ten injections throughout the experiment to monitor the MS performance. To facilitate iRT calculation in Skyline^TM^ for DIA data, protein digests were spiked with the Pierce^TM^ Peptide Retention Time Calibration Mixture (4 fmol/μl), according to the manufacturers’ instructions.

**Discovery Proteomic Analysis using Data Dependent Acquisition (DDA)**

All samples were injected on a Thermo Scientific Q Exactive mass spectrometer connected to a Dionex Ultimate 3000 (RSLCnano) chromatography system. Tryptic peptides (5μl of digest) from each sample were loaded onto a fused silica emitter (75 μm ID, pulled using a laser puller (Sutter Instruments P2000), packed with UChrom C18 (1.8 μm) reverse phase media (nanoLCMS Solutions LCC) and was separated by an increasing acetonitrile gradient over 90 minutes at a flow rate of 250 nL/min. This QC standard was injected 3 times at the beginning of the MS study to condition the column, and after every ten injections throughout the experiment to monitor the MS performance. The mass spectrometer was operated in data dependent TopN 8 mode, with the following settings: mass range 300-1600Th; resolution for MS1 scan 70000; AGC target 3e6; resolution for MS2 scan 17500; AGC target 2e4; charge exclusion unassigned, 1; dynamic exclusion 40 s.

**Targeted Confirmation of Protein Biomarkers using Data Independent Acquisition (DIA)**

The DIA isolation scheme and multiplexing strategy was based on that from Egertson et al. (2013) in which five 4-*m*/*z* isolation windows are analysed per scan [[4](#_ENREF_4)]. Samples were run on the Thermo Scientific Q Exactive mass spectrometer in DIA mode. Each DIA cycle contained one full MS–SIM scan and 20 DIA scans covering a mass range of 490–910^Th^ with the following settings: the SIM full scan resolution was 35,000; AGC 1e6; Max IT 55ms; profile mode; DIA scans were set at a resolution of 17,000; AGC target 1e5; Max IT 20ms; loop count 10; MSX count 5; 4.0 m/z isolation windows; centroid mode [[4](#_ENREF_4)]. The cycle time was 2s, which resulted in at least ten scans across the precursor peak. For DIA library generation, QC samples were injected in DDA mode at the beginning of the run, and after every ten injections throughout the run. The relative fragment-ion intensities, peptide-precursor isotope peaks and retention time of the extracted ion chromatograms from the DIA files were used to confirm the identity of the target molecular species [[4](#_ENREF_4), [5](#_ENREF_5)].

**Bioinformatics and Statistical Analysis**

For DDA, Label-Free Quantification (LFQ), the human FASTA sequence database was searched with MaxQuant (v1.5.2.8) [[6](#_ENREF_6), [7](#_ENREF_7)], as described [[3](#_ENREF_3)]. False Discovery rates (FDR’s) were set to 1% at the peptide and protein level, and only proteins with at least two peptides (one uniquely assignable to the protein) were considered as reliably identified. LFQ intensity values were used for protein quantification between groups. Initial data processing was performed in Perseus (v1.5.0.15), whereby the data was log_2_ transformed to eliminate distributional skew and improve the normal approximation for validity of p-values. Only proteins present in >80% of samples in at least one group were taken forward for quantification, and the filtered data was normalised by subtracting the median intensity for each protein. Statistical analysis was undertaken in RStudio v 0.99.441, following data imputation that was used to replace missing values with a random value from the normal distribution of each protein. Linear regression analysis compared the diagnostic groups including gender and BMI at age 11 as covariates. Fold changes and significance values are reported (p-values and FDR values).

All DIA data was processed in the open-source Skyline software tool (open-source Skyline software tool (<https://skyline.gs.washington.edu>). This tool provided the interface for visual confirmation of protein biomarkers in the samples profiled, without any file conversion. The library was constructed by searching the QC injections, which were interspersed after every ten injections throughout the run, in MaxQuant. As detailed in the online tutorials and publications by the Skyline team, the msms.txt file resulting from the MaxQuant search was used to build the library in Skyline. For our peptide targets, mass chromatograms were extracted for +2 and +3 precursor charge states and their associated fragment ions. Based on our discovery results (Table 2), we targeted 33 complement pathway proteins according to the detailed protocol of Egertson [[5](#_ENREF_5)]. Please see supplementary tables 2a and 2b for the full list of peptides and fragments quantified. For our dataset, the *m/z* tolerance was <10ppm and the average retention time window was 2 minutes. All parent and fragment level data was visually confirmed across the n= 208 samples run, and peak editing was undertaken where necessary, using the peptide Retention Time (RT), dotproduct (idop), mass accuracy (<10ppm), and a confirmed library match to reliably identify and quantify peptides across the DIA runs (Supplementary Figures 1a and b). For statistical analysis, peak areas of the fragment level data was filtered from the Skyline document grid for analysis in mapDIA, an open source bioinformatics tool for pre-processing and quantitative analysis of DIA data[[8](#_ENREF_8)]. Peptide fragment selection using two standard deviation threshold for outlier detection, in the independent sample setup.

**Stress Animal Model**

Male 8 week-old C57BL/6J mice, and 6-month-old CD1 retired breeders, were maintained on a 12h light-dark cycle (lights on at 7 am) at 22-25°C with ad libitum access to food and water. C57BL/6J mice were housed 5 per cage except following defeat experiments at which point mice were singly housed. All experiments were conducted in accordance with the guidelines of the Institutional Animal Care and Use Committee at Mount Sinai. All behavioural testing occurred during the animals’ light cycle. Experimenters were blinded to experimental group and order of testing was counterbalanced during behavioural experiments.

**Chronic Social Defeat Stress (CSDS), behavioural testing and drug treatment**

All experiments utilized an established CSDS protocol to induce depressive-like behaviours in mice [[9](#_ENREF_9), [10](#_ENREF_10)]. All protocols involving mice were approved by the Institutional Animal Care and Use Committee (IACUC) at Mount Sinai School of Medicine. C57BL/6J mice were subjected to 10 daily, 5-min defeats by a novel CD1 aggressor mouse and were then housed across a Plexiglas divider to allow for sensory contact for the remainder of the day. Control mice were housed in cages separated from other control mice by a Plexiglas divider and were rotated to a different cage daily. Social-avoidance behaviour was assessed with a novel CD1 mouse in a two-stage social-interaction test. In the first 2.5-min test (no target), the experimental mouse was allowed to freely explore an arena (44×44cm) containing a Plexiglas and wire mesh enclosure (10×6cm) centered against one wall of the arena. In the second 2.5-min test (target), the experimental mouse was returned to the arena with a novel CD1 mouse enclosed in the Plexiglas wire mesh cage. Time spent in the ‘interaction zone’ (14×26cm) surrounding the Plexiglas wire mesh cage, ‘corner zones’ (10×10cm) and ‘distance travelled’ within the arena were measured by video tracking software (Ethovision 3.0, Noldus).

**Sample Preparation Mass Spectrometry Analysis and Data Processing and Analysis**

Blood was taken 24 hours after social interaction (48 hours after the last defeat), and the plasma/serum was collected by submandibular bleed, allowed to clot at room temperature and then spun at 4°C. Plasma was depleted of the three most abundant proteins, i.e. albumin, IgG, transferrin (Multi Affinity Removal Column Mouse-3, Agilent Technologies, UK).

Protein concentrations were determined and protein was digested and prepared for mass spectrometry as described for the human samples above. Likewise mass spectrometry and data analysis was undertaken as described for the human samples. To avoid bias associated with protein under-representation between groups, proteins were excluded in cases where there was less than 80% availability of the LFQ intensities in each biological group. After data filtering, 704 LFQ values remained.

REFERENCES

1. Levin, Y., et al., *Global proteomic profiling reveals altered proteomic signature in schizophrenia serum.* Mol Psychiatry, 2010. **15**(11): p. 1088-100.

2. Bradford, M.M., *A rapid and sensitive method for the quantitation of microgram quantities of protein utilizing the principle of protein-dye binding.* Anal Biochem, 1976. **72**: p. 248-54.

3. English, J.A., et al., *Reduced protein synthesis in schizophrenia patient-derived olfactory cells.* Transl Psychiatry, 2015. **5**: p. e663.

4. Egertson, J.D., et al., *Multiplexed MS/MS for improved data-independent acquisition.* Nat Methods, 2013. **10**(8): p. 744-6.

5. Egertson, J.D., et al., *Multiplexed peptide analysis using data-independent acquisition and Skyline.* Nat Protoc, 2015. **10**(6): p. 887-903.

6. Cox, J., et al., *Andromeda: a peptide search engine integrated into the MaxQuant environment.* Journal of proteome research, 2011. **10**(4): p. 1794-805.

7. Cox, J. and M. Mann, *MaxQuant enables high peptide identification rates, individualized p.p.b.-range mass accuracies and proteome-wide protein quantification.* Nat Biotechnol, 2008. **26**(12): p. 1367-72.

8. Teo, G., et al., *mapDIA: Preprocessing and statistical analysis of quantitative proteomics data from data independent acquisition mass spectrometry.* Journal of proteomics, 2015. **129**: p. 108-20.

9. Berton, O., et al., *Essential role of BDNF in the mesolimbic dopamine pathway in social defeat stress.* Science, 2006. **311**(5762): p. 864-8.

10. Krishnan, V., et al., *Molecular adaptations underlying susceptibility and resistance to social defeat in brain reward regions.* Cell, 2007. **131**(2): p. 391-404.
