# Supplementary material for: Complement pathway changes at age 12 are associated with psychotic experiences at age 18 in a longitudinal population-based study: evidence for a role of stress
Source: Mol Psychiatry. 2019 Jan 11;26(2):524–33. doi: 10.1038/s41380-018-0306-z (PMC6906256; doi:10.1038/s41380-018-0306-z)
Supplement: Supplementary file 2 — Supplementary Figure 1 [file 41380_2018_306_MOESM2_ESM.pptx]

## Slide 1
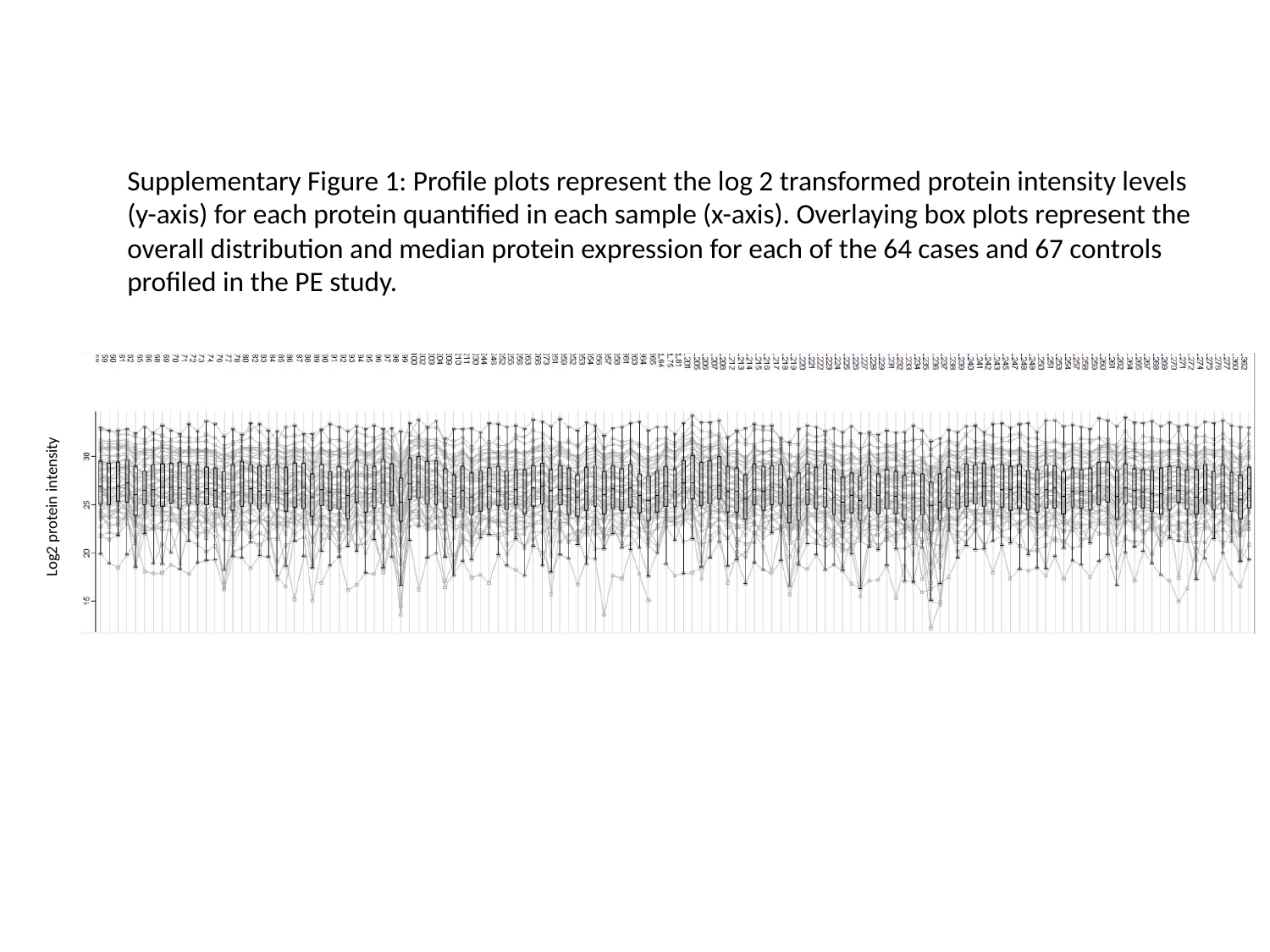

Supplementary Figure 1: Profile plots represent the log 2 transformed protein intensity levels (y-axis) for each protein quantified in each sample (x-axis). Overlaying box plots represent the overall distribution and median protein expression for each of the 64 cases and 67 controls profiled in the PE study.
Log2 protein intensity
