# Supplementary material for: Complement pathway changes at age 12 are associated with psychotic experiences at age 18 in a longitudinal population-based study: evidence for a role of stress
Source: Mol Psychiatry. 2019 Jan 11;26(2):524–33. doi: 10.1038/s41380-018-0306-z (PMC6906256; doi:10.1038/s41380-018-0306-z)
Supplement: Supplementary file 3 — Supplementary Figure 2 [file 41380_2018_306_MOESM3_ESM.pptx]

## Slide 1
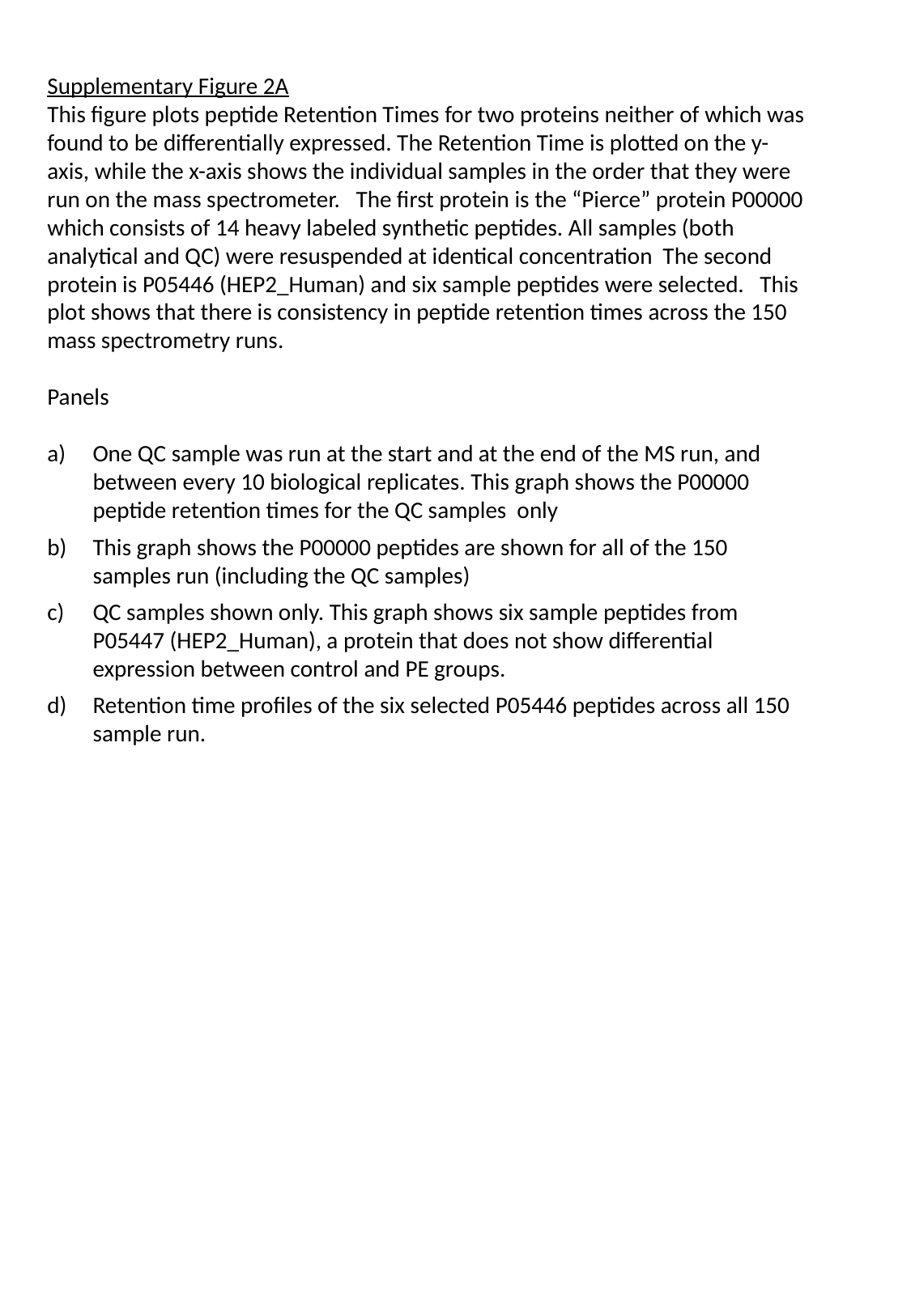

Supplementary Figure 2A
This figure plots peptide Retention Times for two proteins neither of which was found to be differentially expressed. The Retention Time is plotted on the y-axis, while the x-axis shows the individual samples in the order that they were run on the mass spectrometer. The first protein is the “Pierce” protein P00000 which consists of 14 heavy labeled synthetic peptides. All samples (both analytical and QC) were resuspended at identical concentration The second protein is P05446 (HEP2_Human) and six sample peptides were selected. This plot shows that there is consistency in peptide retention times across the 150 mass spectrometry runs.
Panels
One QC sample was run at the start and at the end of the MS run, and between every 10 biological replicates. This graph shows the P00000 peptide retention times for the QC samples only
This graph shows the P00000 peptides are shown for all of the 150 samples run (including the QC samples)
QC samples shown only. This graph shows six sample peptides from P05447 (HEP2_Human), a protein that does not show differential expression between control and PE groups.
Retention time profiles of the six selected P05446 peptides across all 150 sample run.

## Slide 2
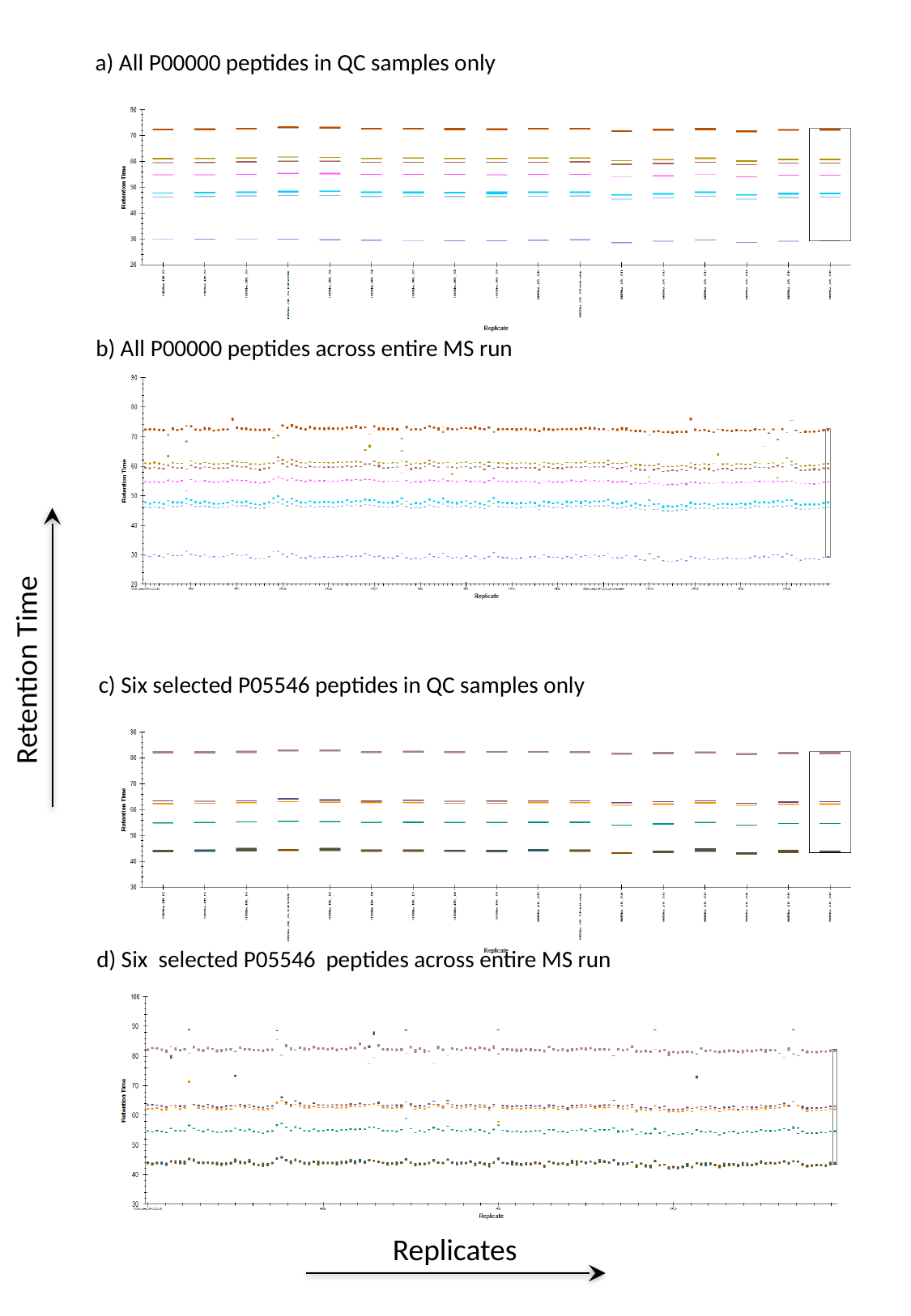

a) All P00000 peptides in QC samples only
b) All P00000 peptides across entire MS run
Retention Time
c) Six selected P05546 peptides in QC samples only
d) Six selected P05546 peptides across entire MS run
Replicates

## Slide 3
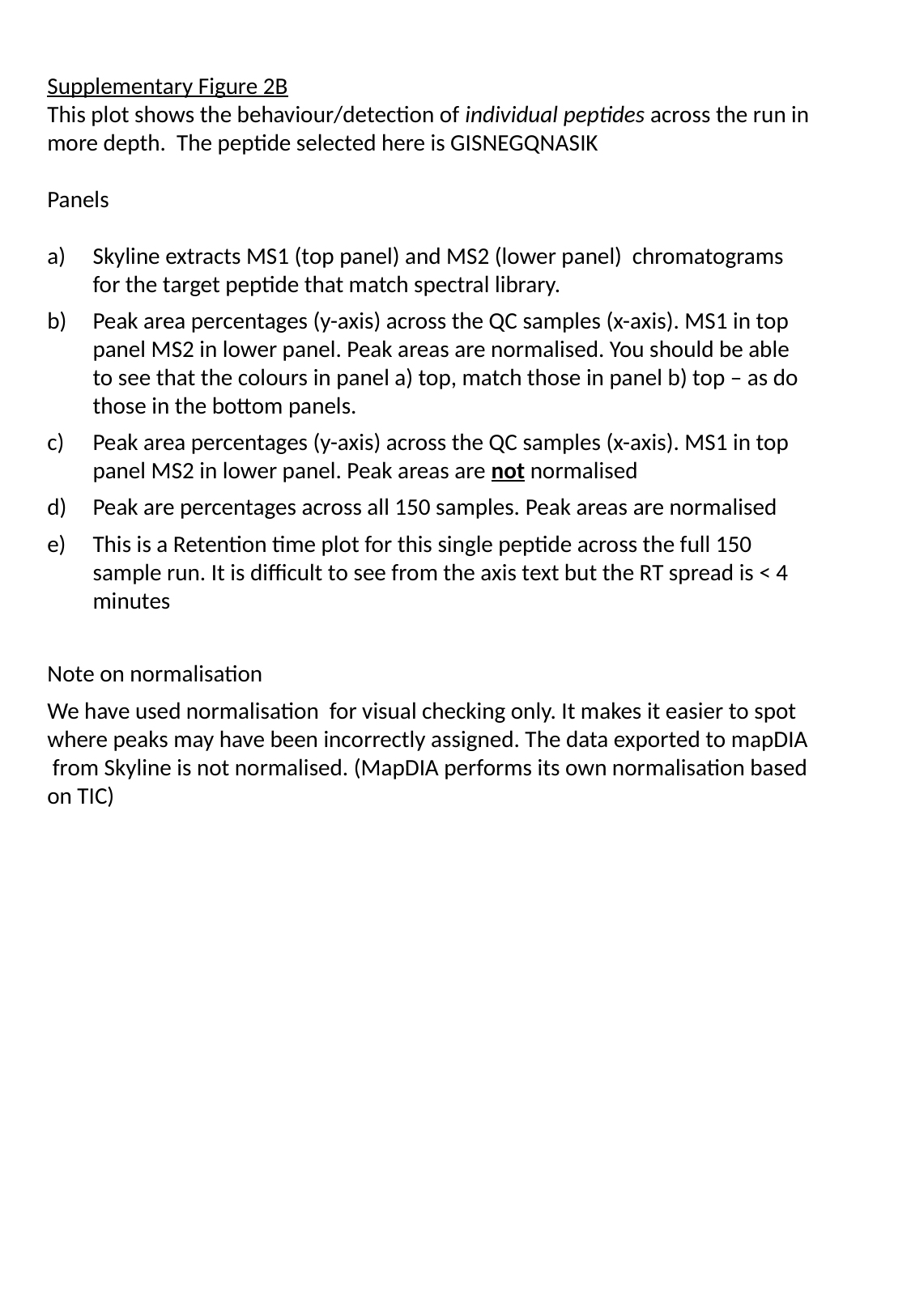

Supplementary Figure 2B
This plot shows the behaviour/detection of individual peptides across the run in more depth. The peptide selected here is GISNEGQNASIK
Panels
Skyline extracts MS1 (top panel) and MS2 (lower panel) chromatograms for the target peptide that match spectral library.
Peak area percentages (y-axis) across the QC samples (x-axis). MS1 in top panel MS2 in lower panel. Peak areas are normalised. You should be able to see that the colours in panel a) top, match those in panel b) top – as do those in the bottom panels.
Peak area percentages (y-axis) across the QC samples (x-axis). MS1 in top panel MS2 in lower panel. Peak areas are not normalised
Peak are percentages across all 150 samples. Peak areas are normalised
This is a Retention time plot for this single peptide across the full 150 sample run. It is difficult to see from the axis text but the RT spread is < 4 minutes
Note on normalisation
We have used normalisation for visual checking only. It makes it easier to spot where peaks may have been incorrectly assigned. The data exported to mapDIA from Skyline is not normalised. (MapDIA performs its own normalisation based on TIC)

## Slide 4
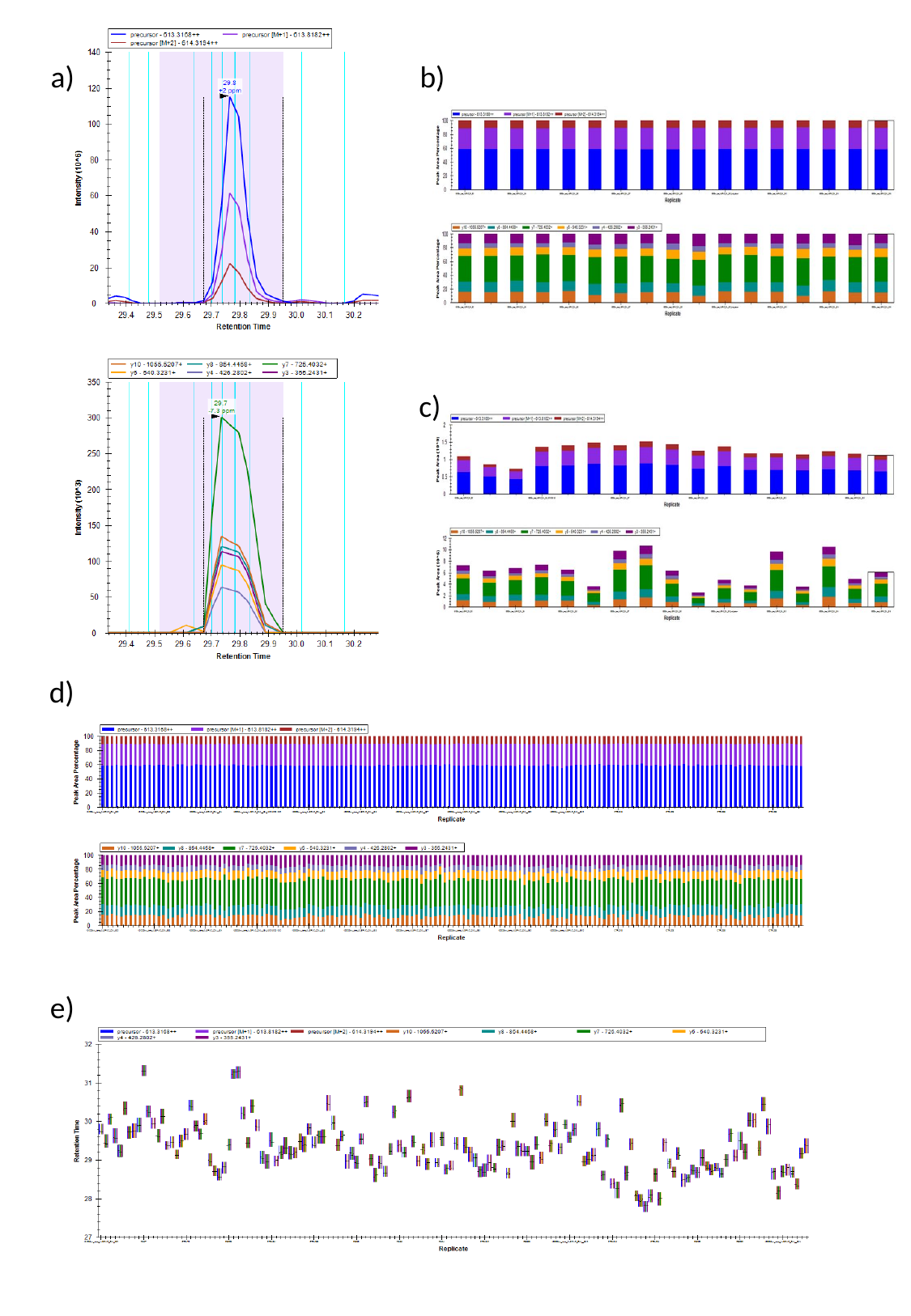

a)
b)
c)
d)
e)
